# Onychomycosis caused by dematiaceous fungi: A four-year study on agricultural workers of Assam, India

**DOI:** 10.22034/cmm.2023.345077.1428

**Published:** 2023-09

**Authors:** Parismita Borgohain, Purnima Barua, Dipika Shaw, Lakhi Ram Saikia, Jagadish Mahanta, Shivaprakash M Rudramurthy

**Affiliations:** 1 Department of Life Sciences, Dibrugarh University, Dibrugarh, Assam, India; 2 Department of Microbiology, Jorhat Medical College, Jorhat, Assam, India; 3 Department of Medical Microbiology, Postgraduate Institute of Medical Education and Research, Chandigarh, India; 4 Regional Medical Research Centre for Northeast, Indian Council of Medical Research, Dibrugarh, Assam, India

**Keywords:** Nail infection, Non-dermatophytes, Phaeoid fungi, Phytopathogens

## Abstract

**Background and Purpose:**

Onychomycosis caused by dematiaceous fungi is rarely reported and the identification is also quite tricky due to poor sporulation. Recent emergence of dematiaceous fungi as a major cause of onychomycosis is a matter of concern in the field of mycology. Therefore, this study aimed to understand the dematiaceous fungi as a possible cause of onychomycosis, especially among agricultural workers. In addition, the evaluation of the antifungal susceptibility patterns led to the idea of an accurate drug that will help to treat and prevent antifungal resistance.

**Materials and Methods:**

The standard procedure was followed for direct microscopic examination and fungi isolation. Furthermore, antifungal susceptibility testing was conducted in accordance with the Clinical and Laboratory Standards Institute M-38-A2 protocol

**Results:**

Both potassium hydroxide and fungal positivity were found in 275 out of 356 suspected cases, 52%, 4.3%, 28.7%, and 14.9% of which were non-dermatophytic molds (NDMs), yeast, dermatophytes, and sterile hyphae, respectively. Among NDMs (52%, n=143), 45.5% (n=65) were hyaline hyphomycetes and 54.5% (n=78) were dematiaceous hyphomycetes. Among dematiaceous fungi, *Pestalotiopsis* spp. and *Arthrinium* spp. were the commonly isolated ones. Additionally, azoles, amphotericin-B, and anidulafungin showed excellent antifungal activity against tested isolates.

**Conclusion:**

Dematiaceous fungi are now becoming a potential cause of onychomycosis. A more detailed study is needed on the identification of these emerging isolates and the mode of action of antifungal drugs for a better treatment strategy.

## Introduction

Onychomycosis is by far one of the most common superficial infections that occur in the general population [ 1 ]. *Trichophyton rubrum*, *T. interdigitale*, *Scopulariopsis brevicaularis*, and *Aspergillus* spp. are involved as primary causative pathogens of onychomycosis. Dematiaceous or phaeoid fungi are a group of non-dermatophytic molds that are rarely implicated in causing onychomycosis [ 2 , 3 ]. This group of fungi produces melanin in cell walls, a brown to black pigment responsible for dark-pigmented colonies, hyphae, or conidia. Melanin has a high molecular weight with a variable molecular structure which is a major enhancing virulence factor reported to cause mild to cutaneous infections in humans [ 4 , 5 ]. *Curvularia*, *Scytalidium*, *Lasiodiplodia theobromae*, and *Exophiala* spp. have been reported in a few cases of onychomycosis [ 1 , 6 ]. However, the identification of the fungi is quite tricky due to colony morphology or poor sporulation [ 3 ]. 

In this regard, the present study aimed to understand the dematiaceous fungi as a possible cause of onychomycosis, especially among agricultural workers. In addition, the evaluation of the antifungal susceptibility patterns led to the idea of an accurate drug that will help to treat and prevent antifungal resistance. 

## Materials and Methods

The present study was conducted in upper Assam, India, in the temperate region between the coordinates 26.5235° N, 93.9679° E and 27.4502° N, 94.8980° E with an average temperature of 26° C. Climatic conditions of upper Assam provide an excellent niche to flourish diverse kinds of flora and fauna. The studied population was categorized into three groups: tea garden workers, rice field workers, and horticulture workers with clinical nail deformities. 

Sensitization programs were conducted among agricultural workers followed by nail sample collection with the help of the managing authority of Tea Estates or The Head of a village. The authors deliberately explained the nail problems prevailing among agricultural groups, which caused discomfort, pain, and aesthetic disfiguration of the nail with the help of pictures, brochures, and information leaflets in the local language. Nail samples were collected after obtaining written informed consent. Moreover, the epidemiological data, namely the demographic characteristics, occupational profiles, and practices were recorded in a pretested questionnaire by ensuring the participants of the confidentiality of their information.

Over a span of four years (from June 2017 to Dec 2021), 356 suspected cases were enrolled in this cross-sectional study. Nail samples were collected following proper cleaning of the affected site with 70% alcohol, utilizing scrapping and/or clipping techniques using nail clippers or blades on clean, dry black paper. Isolate identification relied on the macroscopic and microscopic features of the specimens. Fungal element examination under a microscope involved using 40% potassium hydroxide (KOH) in a moist chamber for 24 h. 

The processing of samples followed the criteria established by Walshe and English. [ 7 ]. Multiple nail pieces were placed in duplicate on Sabouraud dextrose agar (SDA), with and without chloramphenicol (SDAc) (HiMedia), to encourage the growth of dermatophytes, non-dermatophytic molds (NDMs), and yeasts. Positive KOH microscopy in both cultures indicated fungal nail infection, confirmed by repeating the process on additional samples. 

Microscopic identification involved Lactophenol cotton blue mount and slide cultures. The National Culture Collection of Pathogenic Fungi in Chandigarh, India, conducted molecular characterization by sequencing the internal transcribed spacer (ITS)1-5.8S-ITS2 region of the rDNA gene in selected isolates. Genomic DNA extraction followed the phenol-chloroform-isoamyl alcohol method, with subsequent Sanger sequencing [ 8 ]. Amplification of the ITS region occurred in 20-μL reaction volumes using ITS5 and ITS4 primer pairs as described by Prakash et al., 2016 [ 9 ]. 

Polymerase chain reaction sequencing was performed for both strands using the mentioned primers and Big Dye Terminator Cycle sequencing kit, version 3.1 (Applied Biosystems, Foster City, CA, USA). Sequencing products underwent purification and analysis on an ABI Prism 3100 automated DNA analyzer (Applied Biosystems, California, USA). Consensus sequences for each isolate were generated from forward and reverse primer sequences using SeqMan software (version 7.0) [ 8 ], DNASTAR's Laser Gene Genomics, Madison, Wisconsin, USA). 

### Antifungal susceptibility testing of filamentous fungi

*In vitro* antifungal susceptibility testing (AFST) was performed according to the Clinical and Laboratory Standards Institute (CLSI) M-38-A2 broth micro-dilution protocol with *Aspergillus flavus* (ATCC 204304) strain as quality control strain (CLSI M-38-A2) [ 10 , 11 ]. Antifungal drugs, such as Amphotericin B, itraconazole, voriconazole, posaconazole, anidulafungin, caspofungin (Sigma-Aldrich, Bengaluru, India) were used. All drugs were dissolved in dimethyl sulfoxide, whereas caspofungin was dissolved in water. The final concentration of the tested drugs ranged from 0.0312 to 16 µg/ml for amphotericin B, voriconazole, posaconazole, and anidulafungin, and 64 to 0.125 µg/ml for caspofungin. All tested results were sent to medical personnel of respective tea estate hospitals for better treatment of workers.

## Results

In total, 356 clinically suspected cases of onychomycosis were enrolled whose demographic characteristics are presented in Table 1. Regarding gender, 34% (n=121) of the participants were male and the rest were female. Being the most productive and employable age group, the maximum number of participants were in the age group of 21-40 years (59.26%, n=211), followed by 41-60 years (35.39%, n=126), ≤ 20 years (3.37%, n=12), and > 60 years age groups (1.9%, n=7). 

**Table 1:** Demographic characteristics and occupational practices of the participants

Characteristic		Cases	(n=356)
		No.	%
Gender	Male	121	**33.98**
	Female	235	66.01
Age Group range	≤20	12	3.37
	21-40	211	59.26
	41-60	126	35.39
	>60	7	1.96
Types of Occupation	Tea garden worker	156	43.82
	Rice field worker	121	33.98
	Horticulture worker	79	22.19
Nail involvement	Toenails	187	52.52
	Fingernails	169	47.47
Associated condition	Nail pain	356	100
	Irritation	168	47.19
	Nail injury/trauma	67	18.82
	Skin infection	23	6.46
Personal hygiene	Satisfactory	67	18.82
	Poor	289	81.17
Lifestyle exposures	Working with animal excreta	124	34.83
	Household activities	302	84.83
Types of cultivation	Organic	97	27.24
	Inorganic	259	72.75
Associated agricultural habits	Working in a humid moist environment	324	91.01
	Walking barefooted	345	96.91
Duration of occupation	≤5 years	65	18.25
	>5 to 10 years	102	28.65
	>10 years	189	53.08

Majority of nail samples were collected from toenails (187/356, 52.52%), while 169/356 (47.47%) were from fingernails. Their chief complaints were pain (n=356, 100%) and irritation (n=168, 47.19%). Most of the participants were involved with inorganic cultivation practices (n= 259, 72.7%) and long duration of occupation (>10 Years, n=189, 53.08%).

**Figure 1:**
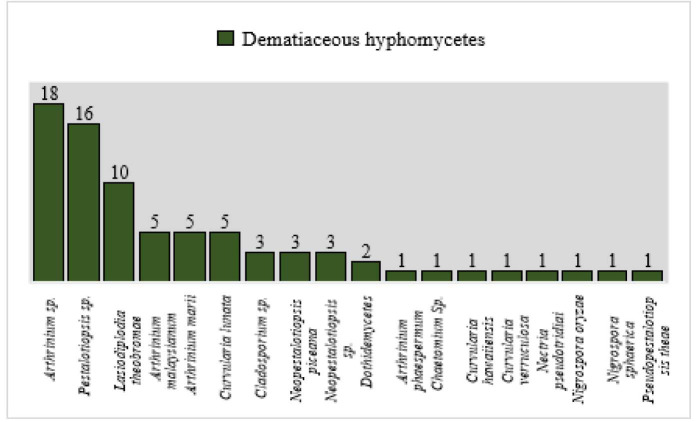
Dematiaceous hyphomycetes isolated from onychomycosis cases (n=78)

**Figure 2.1:**
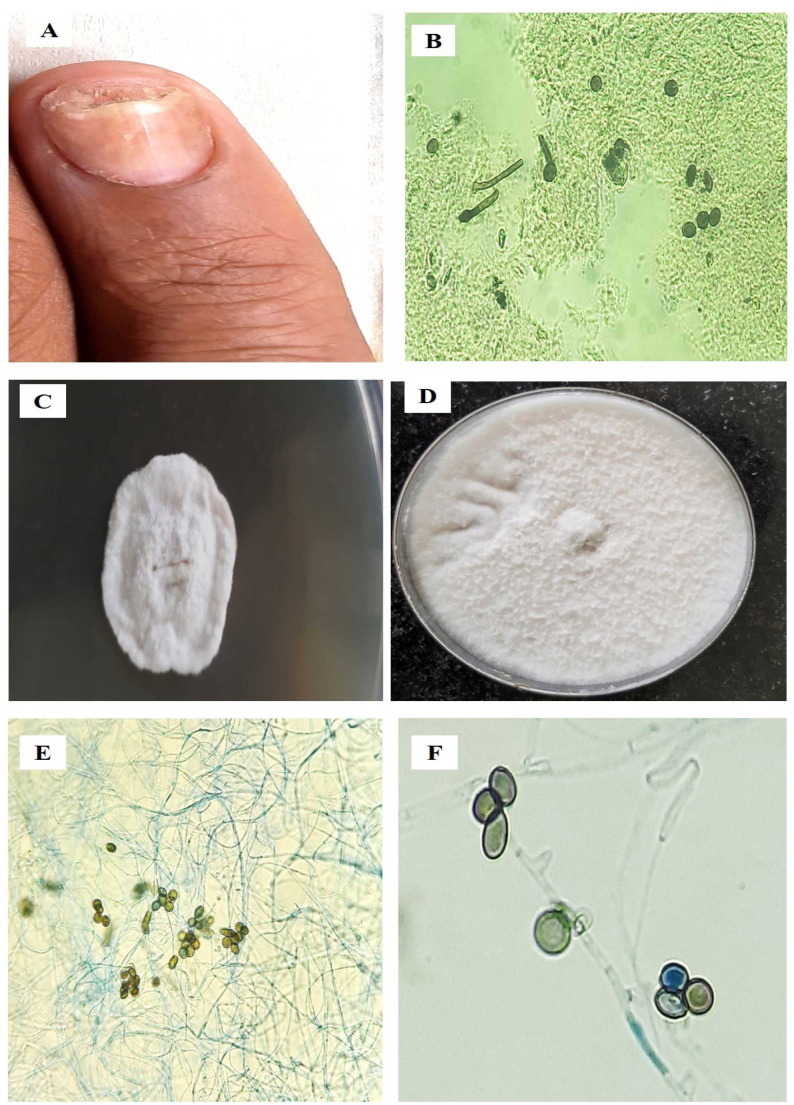
***Arthrinium marii* (MK926438)**. A. Distal lateral subungual onychomycosis, B. Dematiaceous septate hyphae, C. White cottony colony on SDAc (7 days), D. Mature fungal culture, E and F. Globose conidial morphology

**Figure 2.2:**
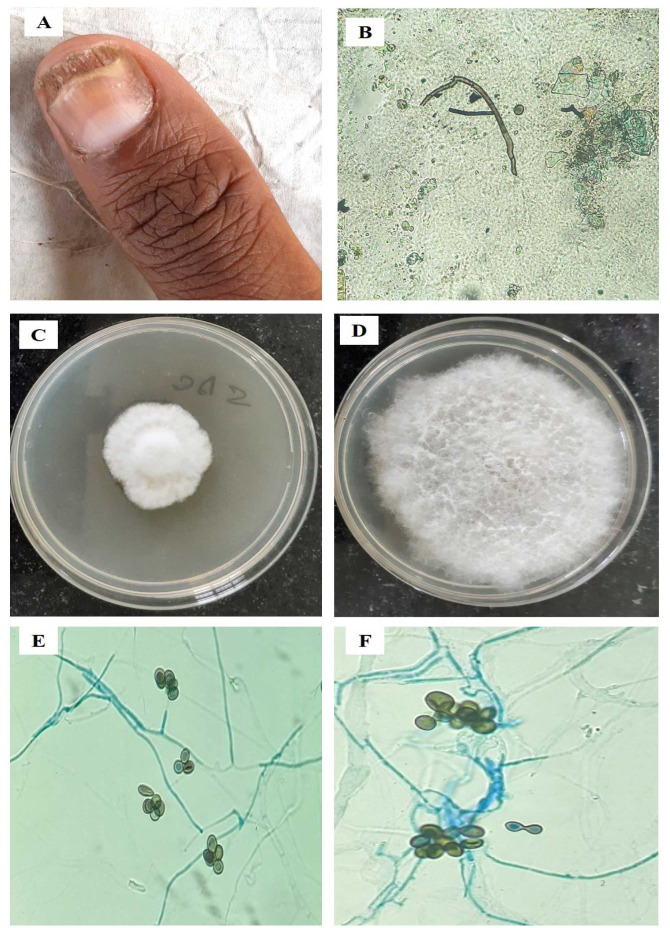
***Arthrinium* sp. (MT672554)** A. Distal lateral subungual onychomycosis, B. Dematiaceous septate hyphae, C. White cottony colony on SDAc (7 days), D. Mature fungal culture, E and F. Globose conidial morphology

**Figure 2.3:**
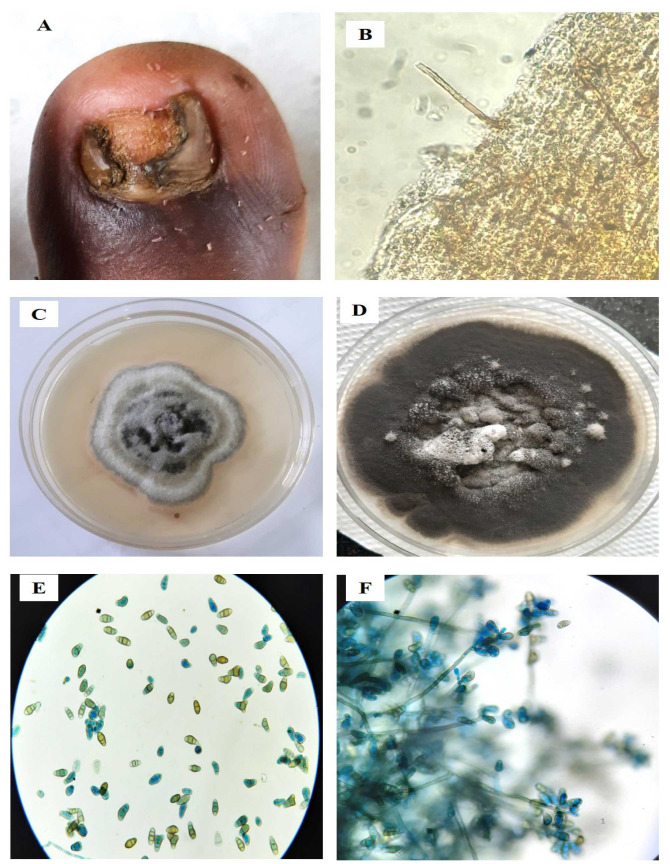
***Curvularia lunata* (MT672526)**. A. Total Dystrophic Onychomycosis, B. Potassium hydroxide mount showing dematiaceous septate hyphae, C. Light grey cottony colony on SDAc (7 days), D. Mature fungal culture, E. Conidia with distinct curved shape with narrower septation between cells, central cells of conidia darker than the end cells, F. Microscopic morphology

**Figure 2.4:**
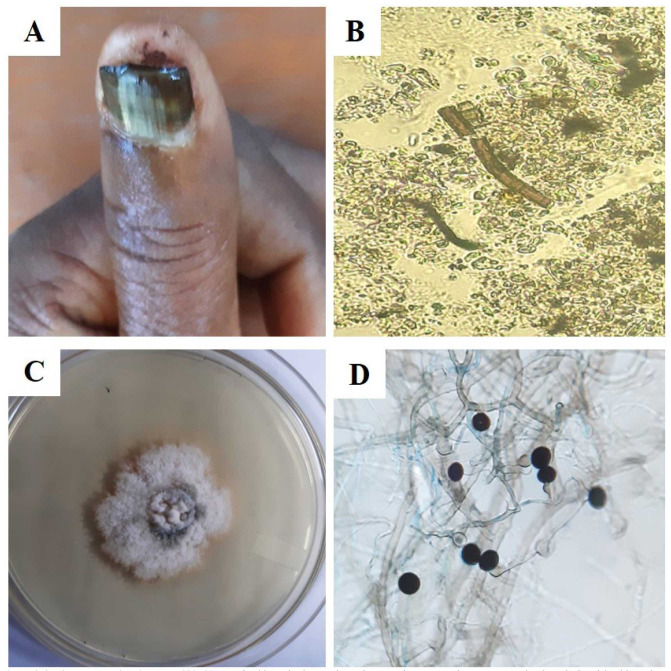
***Nigrospora sphaerica* (MW644541)**. A. Distal lateral subungual onychomycosis, B. Dematiaceous septate hyphae, C. Grayish white colony on SDAc in 7 days of culture, D. Sporulation of *Nigrospora sphaerica* and large densely black conidia attached to their short conidiophores

The KOH and culture positivity were found in 275 cases (77.2%) out of 356 clinically diagnosed onychomycosis cases. On culture, 52% (n=143), 4.3% (n=12), 28.7% (n=79), and 14.9% (n=41) were NDMs, yeast, dermatophytes, and sterile hyphae, respectively (Figure 1). Most of the agricultural workers had NDM infection (n=143, 52%) on the fingernails (55.2%, n=79), compared to toenails (44.7%, n=64). Among NDMs, 45.4% (n=65) were hyaline hyphomycetes and 54.5% (n=78) were dematiaceous hyphomycetes. Moreover, among the dematiaceous fungi (54.5%, n=78), *Arthrinium* sp. (23%, n=18) [Figure 2.1 & Figure 2.2], *Pestalotiopsis* sp. (20.5%, n=16), *L. theobromae* (12.8%, n=10), and *Curvularia lunata* (6.4%, n=5) [Figure 2.3] were frequently isolated species (Figure 1). Rarely identified isolates were *Nigrospora oryzae*, *Nigrospora sphaerica* [Figure 2.4], *Nectria pseudotrichia*, and *Dothidemycetes*.

In this study, 29 ITS sequences of dematiaceous fungi were deposited in GenBank National Center for Biotechnology Information under the following accession numbers presented in Table 2.

**Table 2:** Identified isolates with their accession numbers.

Identified isolates	GenBank accession number
*Arthrinium malaysianum*	MK926440, MK926439, MK926437, MT672528, MT672557, MT672561
*Arthrinium marii*	MK926438 (Figure 2.1), MT672553, MT672555, MT672558, MT672560, MW644534, MW686904, MW686905
*Arthrinium* sp.	MT672554 (Figure 2.2)
*Pestalotiopsis* sp.	MT672527, MT672529
*Neopestalotiopsis piceana*	MT672559
*Curvularia hawaiiensis*	MN006200, MN006199
*Curvularia verruculosa*	MN068858
*Lasiodiplodia theobromae*	MT672562, MW644538
*Nectria pseudotrichia*	MN078198
*Dothidemycetes*	MN078200
*Arthrinium phaeospermum*	MT672556
*Curvularia lunata*	MT672526 (Figure 2.3)
*Nigrospora oryzae*	MW644540
*Nigrospora sphaerica*	MW644541 (Figure 2.4)

### In vitro antifungal activity

The antifungal susceptibility pattern of dematiaceous hyphomycetes was detected to be susceptible to tested drugs with varied minimum inhibitory concentrations (MICs). *Arthrinium phaeospermum* had a low MIC value (0.0312 µg/mL) for amphotericin B, anidulafungin, and posaconazole. All species of *A. marii* (n=5) were susceptible to voriconazole and amphotericin B with MICs of 0.0312 µg/ml. Majority of the *Curvularia* spp. had low MIC values for posaconazole which ranged from 0.0312 to 0.25 µg/mL. Posaconazole and caspofungin showed the lowest MIC values for *C. hawaiensis* (n=1) which was 0.0312 µg/mL. Moreover, posaconazole was observed to be inhibiting *C. lunata* (n=3) effectively at 0.625 µg/mL, and *C. verruculosa* (n=1) showed the lowest MIC value at 0.0312 µg/mL for all the tested azoles.

*Pseudopestalotiopsis theae* (n=2) showed the least MIC values for voriconazole, and posaconazole (0.0312 µg/mL), while *Pestalotiopsis theae* (n=4) and *Neopestalotiopsis* sp. (n=3) showed the maximum susceptibility to anidulafungin at 0.0312 µg/mL among the drugs tested. The AFST results against all tested isolates were presented in Supplementary Table 1. 

## Discussion

The present study depicted the emergence of dematiaceous fungal isolates as a causative agent of onychomycosis among agricultural workers. *Arthrinium* spp., *Pestalotiopsis* spp., and *Curvularia* spp. were frequently identified as the causative agents in this study. Studied group of the population was constantly engaged in agricultural practices for their livelihood in harsh environmental conditions. Directly handling inorganic cultivation practices (n=259, 72.7%), habit of walking barefooted (n=345, 96.91%), duration of occupation of more than 10 years (n=189, 53.08%), and severe nail pain (n=356, 100%) could be the associated inducing factors of onychomycosis. 

Various studies have reported exposure to mud, cow dung, manure, fertilizers, herbicides, pesticides, and harvesting practices were the predisposing factors for fungal infection among farmers [ 13 - 16 ]. Barua et al. [ 17 ] and Toukabri et al. [ 18 ] reported that walking barefooted is a risk factor for nail fungal infection due to direct contact with the soil and that practicing sports with ill-fitting shoes increases trauma of the nail. Pierard [ 19 ] and Scher and Baran [ 20 ] noted that the long duration of occupation represents longer exposure to pathogenic fungi, larger and distorted nail surfaces, and repeated nail trauma. 

Additionally, most of the reported fungal isolates from this region were opportunistic non-dermatophytes [ 3 , 12 ]. Conidia of these molds dispersed through biotic factors, developed fungal diseases in agricultural fields, and then transmitted to humans. These fungal strains have gained attention not only for their role as phytopathogens, but also for their increasing presence in human ailments [ 12 ]. Specifically, *L. theobromae*, known for its limited sporulation, was found to be resistant to the commonly used antifungal treatments. The same authors have previously documented three cases where *L. theobromae* was identified in the deformed nails of agricultural workers [ 3 ]. 

Pestalotioid fungi are typically found in environmental settings and are not commonly associated with human infections [ 12 , 21 , 22 ]. However, 16 instances of these fungi were isolated from the infected nails of agricultural workers. *Arthrinium* spp., known for its varied ecological roles and often found as an endophyte, has been recognized as a plant pathogen causing Leaf Blight in tea plants [ 23 ]. Through morphological characterization and genetic analysis using the ITS regions of rDNA, four species were identified within the *Arthrinium* genus- *A. malaysianum*, *A. marii*, *Arthrinium* sp., and *A. phaeospermum*-signifying the connection between environmental molds and onychomycosis.

*Curvularia* species are known for their significance as plant pathogens and occasional human pathogens [ 24 , 25 ]. Molecular identification of *Curvularia* using the ITS region is a common practice for distinguishing species and understanding variations among them [ 26 , 27 ]. While onychomycosis caused by *Curvularia* sp. is seldom reported, three clinically important species we identified, namely *C. hawaiiensis*, *C. verruculosa*, and *C. lunata*, all of which belong to the group of dematiaceous fungi known for the production of melanin pigments [ 28 ].

Nail infections by *Curvularia* sp. have been documented among farmers by Vijaya et al. [ 29 ] and Vineetha et al. [ 30 ]. However, infections, specifically those caused by *C. hawaiiensis* and *C. verruculosa* remain uncommon. However, *C. lunata*, dispersed through its airborne spores, is widely prevalent and is a frequent cause of plant diseases [ 31 ]. In the present investigation, two species of *Nigrospora* spp., namely *N. oryzae* and *N. sphaerica*, were identified. Typically recognized as a plant pathogen and an endophyte, *Nigrospora* spp. infrequently leads to human infections [ 32 ]. Fan et al. [ 33 ] previously documented the inaugural case of onychomycosis in humans caused by *N. sphaerica*. To the knowledge of the authors, the present study marks the first instance of onychomycosis attributed to *N. oryzae*.

*Nectria*, a plant pathogen belonging to the Ascomycete fungi, has not been previously associated with causing onychomycosis. However, the present study presents the first isolation of *N. pseudotrichia* from the toenail of a female worker. *Nectria* species are commonly found as saprophytes on decaying wood, typically causing canker and twig dieback diseases, particularly in hardwood trees, like the *Camellia* plant [ 24 ].

While filamentous fungal infections have been on the rise in India, there is a lack of a comprehensive evaluation of antifungal susceptibility, particularly in dematiaceous fungi. In the present study, among the Pestalotioid group fungi, *Pseudopestalotiopsis theae* displayed the lowest MIC values for voriconazole and posaconazole (0.0312 µg/mL). *Pestalotiopsis theae* and *Neopestalotiopsis* sp. showed the highest susceptibility to anidulafungin at 0.0312 µg/mL among the tested drugs. A PubMed search revealed no reported antifungal tests against the Pestalotioid group causing onychomycosis. 

Gajjar et al. [ 34 ] reported good *in vitro* activity of amphotericin B and natamycin against *C. lunata*. In this study, posaconazole exhibited the best MIC value (0.625 µg/mL) against *C. lunata*. For *C. verruculosa*, itraconazole, posaconazole, voriconazole, and amphotericin- B displayed the lowest MIC values (0.0312 µg/mL). Azoles are known for targeting the fungal cell wall and inhibiting the C14α demethylation of lanosterol, which destabilizes cells by depleting the synthesis of ergosterol in the cell membrane [ 35 ]. Itraconazole is the only FDA-approved antifungal drug against NDMs since it strikes a balance between potency and safety in terms of pharmacokinetic properties. 

Fluconazole is not FDA-approved for the treatment of onychomycosis; however, it is utilized off-label by healthcare professionals [ 36 ]. Novel antifungal treatment is the need of the hour to reduce morbid conditions, combat toxicity, and overcome safety and drug resistance challenges to improve the prognosis of onychomycosis.

## Conclusion

A wide range of phytopathogens from agricultural fields is now being recognized as a potential threat to onychomycosis. Accurate laboratory isolation and identification of emerging dematiaceous environmental molds is the need of the hour to understand changing the mycological scenario based on the occupational risk of the agricultural community.
